# Real-time recommendations for energy-efficient appliance usage in households

**DOI:** 10.3389/fdata.2022.972206

**Published:** 2022-09-20

**Authors:** Magdalini Eirinaki, Iraklis Varlamis, Janhavi Dahihande, Akshay Jaiswal, Akshay Anil Pagar, Ajinkya Thakare

**Affiliations:** ^1^Computer Engineering Department, San José State University, San Jose, CA, United States; ^2^Department of Informatics and Telematics, Harokopio University of Athens, Athens, Greece

**Keywords:** real-time recommendations, sequential rule mining, disaggregation, load monitoring, energy efficiency, recommender systems

## Abstract

According to several studies, the most influencing factor in a household's energy consumption is user behavior. Changing user behavior to improve energy usage leads to efficient energy consumption, saving money for the consumer and being more friendly for the environment. In this work we propose a framework that aims at assisting households in improving their energy usage by providing real-time recommendations for efficient appliance use. The framework allows for the creation of household-specific and appliance-specific energy consumption profiles by analyzing appliance usage patterns. Based on the household profile and the actual electricity use, real-time recommendations notify users on the appliances that can be switched off in order to reduce consumption. For instance, if a consumer forgets their A/C on at a time that it is usually off (e.g., when there is no one at home), the system will detect this as an outlier and notify the consumer. In the ideal scenario, a household has a smart meter monitoring system installed, that records energy consumption at the appliance level. This is also reflected in the datasets available for evaluating such systems. However, in the general case, the household may only have one main meter reading. In this case, non-intrusive load monitoring (NILM) techniques, which monitor a house's energy consumption using only one meter, and data mining algorithms that disaggregate the consumption into appliance level, can be employed. In this paper, we propose an end-to-end solution to this problem, starting with the energy disaggregation process, and the creation of user profiles that are then fed to the pattern mining and recommendation process, that through an intuitive UI allows users to further refine their energy consumption preferences and set goals. We employ the UK-DALE (UK Domestic Appliance-Level Electricity) dataset for our experimental evaluations and the proof-of-concept implementation. The results show that the proposed framework accurately captures the energy consumption profiles of each household and thus the generated recommendations are matching the actual household energy habits and can help reduce their energy consumption by 2–17%.

## 1. Introduction

Global energy usage is on the rise with no indications of slowing down. With the increase in standards of living, the domestic household has seen an unprecedented increase in electricity demand. The EIA (US Energy Information Administration) predicts that energy consumption in the building sector (i.e. commercial and residential structures), would rise by 65% between 2018 and 2050 (Shrestha, 2020). Electricity consumption in the residential sector constantly rises as income and standard of life rise. According to the Residential Energy Consumption Survey (RECS) from 2015, 31% of U.S. households have difficulty paying their electricity bill or maintaining a comfortable home temperature with 20% of the households choosing to reduce or forgo necessities such as food or medicine to pay electricity bills (Administration, 2018). The recent Annual Energy Outlook (AEO) (Center, 2020) released by the U.S. Energy Information Administration foresees an increase of 1% per year to the annual electricity use, which projected to 2050 results to an increase in the residential sector from 1,500 to 1,900 billion kWh.

In order to mitigate this problem, there has been an increasing interest in the development of smart energy-saving solutions. Researchers are learning about the most influential aspects in energy consumption patterns in parallel to their study on smart energy-saving technologies. Existing smart home solutions come with monthly summaries of energy use by appliance, but this is insufficient to encourage consumers to maintain a long-term energy-saving behavior. Given that the energy consumed in a household is mainly dominated by construction-related factors, which demand expensive transformations to achieve energy efficiency, user behavior seems to be the most easily changeable variable (Gavalas and Kenteris, 2011; Baltrunas et al., 2012; Savage et al., 2012; Sardianos et al., 2019; Starke et al., 2020). In particular, it has been shown that giving direct and real-time feedback to the customer on their energy consumption has a much better effect than monthly bills or weekly recommendations in terms of encouraging energy conservation (Abrahamse et al., 2005). In addition, building repetitive energy-saving activity patterns can assist in forming better habits and consequently to reduce energy consumption (Gram-Hanssen, 2013). This has been our motivation in this work. Providing real-time recommendations to save energy and change user habits into efficient energy utilization behavior is the key to solve this problem. However, one must find a balance between behaviors that make sense and those that depart significantly from the user's regular behavior, as the latter may result in a poor user acceptance rate (Starke et al., 2017).

In the ideal scenario, a household has a smart meter monitoring system installed, that records energy consumption at the appliance level. This is also reflected in the datasets available for evaluating such systems (Kelly and Knottenbelt, 2015; Sun et al., 2019). Although this method provides a systematic, comprehensive, and convenient way of collecting data, it still has shortcomings. Intrusive load monitoring is complicated to implement and maintain, is not always cost-effective, and faces problems with customer acceptance since customers do not like the intrusion of their privacy. As a result, in the common scenario the household has only one main meter and not separate consumption meters for each appliance. These non-intrusive load monitoring (NILM) techniques use this single monitor and data mining techniques to estimate the consumption of each appliance (Aladesanmi and Folly, 2015; Jiang et al., 2019; Nalmpantis and Vrakas, 2019). In NILM, the main meter reading data is acquired as an input, and then is disaggregated to obtain power consumption of each appliances present in a house. It is a low cost solution without the interference of third-party monitoring devices into the customer's household while monitoring energy consumption data.

**Figure 1:**
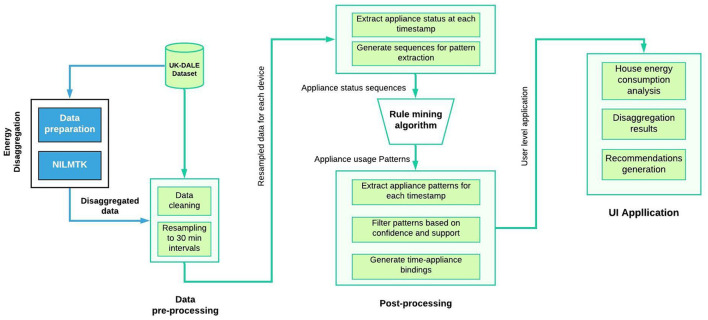
The main processing flow of the proposed framework.

In this work, we focus on the entire process as applicable to most households, including the energy disaggregation step. We present a framework that learns a household's energy usage habits and provides real-time recommendations for energy conservation throughout the day. We extend our previous work (Dahihande et al., 2020) that focused solely on the recommendation algorithm and offered a glance of the UI, by providing more background, technical details, and experimental results spanning the entire process, from disaggregation to post-processing, giving a holistic view of the proposed framework. The system architecture, depicted in Figure 1, includes offline modules for preprocessing the single meter data, disaggregating the readings per appliance, and mining frequent appliance usage patterns, and a real-time module that employs an interactive user interface to send energy saving recommendations.

To support our claim that NILM techniques can be combined with recommender systems in order to reduce domestic energy consumption and in an attempt to identify the most accurate energy disaggregation method, we compare various NILM techniques on the UK-DALE dataset (Kelly and Knottenbelt, 2015). The main idea behind disaggregation methods is to recognize transitional changes in the power consumption, and associate these changes to the respective appliance that is causing it. This is a process that analyzes the level of change and associates it with the energy footprint of one or more appliances. We ran an experimental evaluation on various disaggregation methods, but first resampled the raw data at 2 min intervals, in order to rectify anomalies in the time periods recorded. We observed that the Factorial Hidden Markov Model (FHMM) performed better for energy disaggregation for the UK-DALE dataset (as measured by RMSE, and precision/recall/F1).

The pattern mining module is the heart of the proposed recommender system and is used to extract appliance usage profiles. The profiles retrieved from the appliance-level data are considered as the standard user behavior, since we don't have any other explicit set of optimum electricity usage. So when outliers are detected, the engine generates personalized recommendations on which appliances must be turned on/off, which are communicated with the user through a UI. For example, if an appliance is turned on at a time that differs from the user's appliance usage pattern (e.g., the coffee machine is turned on at 11 p.m.), the user will receive a recommendation to turn it off. This approach allows lowering the consumers' daily power use and optimizing energy utilization. The prototype that we designed and implemented is a proof-of-concept (POC), evaluated on the UK-DALE dateset, that shows how personalized recommendations may vary depending on the user/household and the time of day.

Several association rule mining algorithms, such as *Apriori* and FP-Growth, and sequential pattern mining methods, such as CMRules, RuleGrowth, ERMiner, and CMDeo, have been considered for extracting interesting appliance usage patterns form the UK-DALE dataset. The nature of the data, which consists of detailed power consumption readings at a few seconds' intervals, recorded for several months, present many challenges for the pattern mining algorithms. Data must be properly preprocessed and resampled in order to get useful and interpretable patterns that can be the basis for generating recommendations. As shown in the experiments, the resulting recommendations captured most of the household's appliance usage habits with a high recall, and could have helped in reducing the households' energy consumption from 2 to 17% if implemented (Dahihande et al., 2020). The discovered patterns are also empirically verified using 2D plotting with the help of t-SNE, that captures the time/appliance associations.

In a nutshell, the contributions of this work are summarized in the following:

- A comparison of non-intrusion monitoring techniques and disaggregation algorithms.
- A methodology for extracting appliance usage patterns from household consumption data.
- A recommender system that uses house energy consumption profiles to generate energy-saving recommendations, prompting users to turn off appliances.
- A proof-of-concept application that visualizes consumption, allows to control devices and delivers real-time recommendations.

The rest of the paper is organized as follows: In Section 2, we review the various models used for energy disaggregation and energy consumption pattern extraction, and list the main datasets used for experiments in the related works. Section 3 details on non-intrusive load monitoring techniques that we have implemented in this study and Section 4 describes the approach followed for extracting appliance usage patterns. Section 5 illustrates our proof-of-concept implementation and discusses its main features. In Section 6, we discuss the experimental evaluation setup and present the most important findings. Finally, Section 7 concludes the paper with our plans for extending this work on recommender systems for energy efficiency. Throughout the paper, we use data from UKDALE's House 2 energy readings as a running example.

## 2. Related work

### 2.1. Energy disaggregation models

Non intrusive load monitoring (NILM) is a practical way of analyzing energy consumption and the state of operation of individual appliances based on the main meter reading. In NILM we estimate which appliances are being used, and calculate the energy consumption of individual appliances by analyzing voltage and power reading fluctuations in main power. It is called non-intrusive since it does not require any third-party device.

Aladesanmi and Folly (2015) proposed an approach for non-intrusive load monitoring to identify the appliance's electrical consumption. In their approach, every appliance's energy signature is calculated and plotted on a 2D plane, and once this is done, K-means was used to classify each moment to the appliance that is currently turned on. However, this approach was not able to detect continuous variable appliances, such as light dimmers. He et al. (2016) proposed a graph signal processing approach to distinguish between the appliances that evoke a measured aggregate active power at any moment. Again, this method assumes only two-state appliances (on/off).

Altrabalsi et al. (2016) pointed out that NILM techniques (note that in their paper they call them “NALM”) that can disaggregate power loads at low sampling rates are not accurate enough and require substantial input and long training periods. They proceed to propose two approaches based on a combination of K-means and SVM. They used a house-agnostic training data from a database of over 200 house appliance signatures for training and compared it to house-specific training data, a comparison that highlighted the trade-off between accuracy and complexity.

Jiang et al. (2019) investigate the problem of energy disaggregation together with the problem of the appliance on/off detection. Experiments with the recently proposed WaveNet model for energy disaggregation conclude that WaveNet is better at handling long sequences and it outperforms the previous works based on CNN and RNN models. They also study the performance of two learning frameworks, regression-based, and classification-based. Classification based learning model performs better in terms of F1 scores.

Sadeghianpourhamami et al. (2017) suggest that the effectiveness of the Non-Intrusive Load Monitoring algorithm to identify various electrical appliances depends upon the selection of discriminative features. Nalmpantis and Vrakas (2019) extensively discuss 10 different algorithms that were compared using various factors. While evaluating these NILM systems, the authors concluded that there is a lack of metrics for the majority of requirements and it is suggested to use simple metrics. According to the authors, neural networks show the promise of meeting the requirements of generalization.

Liu et al. (2021) were also based on motif discovery to mine appliance power consumption patterns for the first time, and explored the possibility of applying the same method in unsupervised NILM. Codispoti et al. (2022) introduced the K-Active-Neighbors (KAN) algorithm that jointly learns the user behavior and the appliance signatures, by asking the user feedback when necessary. The method requires the user availability in order to improve the confidence score of the detected signatures.

Himeur et al. (2021a) propose a non-intrusive load monitoring scheme that is based on 2D phase encoding of power signals. The authors extract time-domain (TD) features of the power signal using sliding windows, then take a two-dimensional representation of the signal, aiming to encode more power features than 1D representations, and finally apply a local phase encoding process on the frequency representation of the obtained matrix using a block splitting method. The histogram of the 2D phase encoding of power signals (2D-PEP) is generated by converting the binary codes to decimal representations and various classifiers, such as k-nearest neighbors (Himeur et al., 2021b) and ensemble bagging tree (Himeur et al., 2020), are tested in order to detect the appliance behind each on/off event.

Deep neural networks, and more specifically 1D CNN combined with MLPs, have been employed by Faustine et al. (2020) in UNet-NILM for detecting the state and estimating the power consumption of various appliances, as a multi-task learning problem. The approach was based on the UNet architecture, initially proposed for image segmentation, and a multi-label classification technique. Although NILM systems have significantly progressed in the task of disaggregation, when they are employed in the problem of energy efficiency, they do not provide appropriate attention to the context in which the users interact with the appliances, which is of utmost importance in the case of recommender systems.

Murray et al. (2019) have also proposed two neural network architectures (a GRU and a CNN) for processing low rate measurements (1–60 s sampling rate), and detecting the appliance on/off state or predicting its consumption, respectively. They trained one model for each target appliance and tested the transferability of the models across different NILM datasets. In a similar task, Zhao et al. (2020) tested Factorial Hidden Markov Models, graph signal processing, CNN, and other techniques and concluded that CNNs our perform all other methods. They also found that unsupervised methods, which are based on manufacturer information about consumption, estimation and removal of the baseload, can be comparable in efficiency.

Sardianos et al. (2019) approach the energy disaggregation problem using K-means centroids. The authors design combinations for all available appliances in a room based on the wattage of an appliance. Then using K-means they find centroids equal to the number of combinations of appliances and associate the voltage power change to a respective combination. Although this approach is impressive, it assumes most of the appliance combinations and their energy consumptions and generates a large number of clusters.

Kaselimi et al. (2020b) introduce a non-causal adaptive context-aware bidirectional deep learning model for energy disaggregation, which combines LSTM networks, with non-causality and adaptivity to context (e.g., seasonality). The same authors have also proposed a Generative Adversarial Network (GAN) approach which is capable of making more long-term estimations (Kaselimi et al., 2020c). Their approach was tested mainly in terms of predicting the amount of power consumed by each appliance, although it could be used for detecting on/off actions. Finally, in Kaselimi et al. (2019) they propose a CNN-based architecture that incorporates past estimation outputs and is robust to noise, and in Kaselimi et al. (2020a) they combine GANs with a CNN in the discriminator for rapid processing and optimal extraction of features. The latter method further improves the performance of Kaselimi et al. (2020c) in predicting the appliances' consumption.

### 2.2. User energy consumption pattern extraction

Understanding appliance usage trends is the first stage in developing energy-saving suggestions for users. Without having to search the complete dataset for frequent itemsets, Singh and Yassine (2019) were able to extract inter-appliance relationships more quickly. For this purpose, they used association rule mining and incremental frequent pattern extraction techniques. The extracted itemsets, however, include the combined use of appliances and not associations of individual appliances with time that show which appliances are on or off at specific times throughout the day.

Ong et al. (2013) have collected data from 53 plug-based meters for 3 months and they detected on and off events using only frequency and voltage measurements. A post-processing of these events, with the use of sequential rule mining algorithms, such as *Apriori*-Inv, FPGrowth, and TRuleGrowth lead to the extraction of interesting usage patterns. Setting a confidence threshold above 0.7 and various support thresholds they found patterns which are either combos of appliances used together, such as TV with speakers, or sequentially, such as coffee machine used after toaster. Using smaller support values they found usage patterns comprising rare devices, which have to be further analyzed.

Processing the retrieved patterns is challenging since the number of obtained rules can be huge when very low support thresholds are employed. Additionally, because the patterns were not associated with specific time-zones within the day, they cannot be used directly to provide recommendations during the day.

Another group of works, focuses on the analysis of smart meter data for the extraction of energy consumption profiles (Sial et al., 2014) and the detection of outlying and abnormal behaviors (Sial et al., 2018, 2021). Such abnormalities can be the triggers for recommendations or alerts to the user in order to reduce energy.

In an attempt to design real-time recommender systems that provide energy-efficient recommendations based on various contexts, Sardianos et al. (2019) and Alsalemi et al. (2020) propose a recommendation engine that attempts to shape the daily habits of users and help them lower their energy footprint. Their engine examines various contexts and offers recommendations for actions that promote energy-saving. In order to detect when the monitored appliances are used from a single meter data they apply energy disaggregation algorithms. Then they extract utilization patterns at the appliance level using the *Apriori* algorithm with a support threshold of 0.02, which corresponds to more than 12 appliance usages per month. The resulting recommendations are based on general energy-saving rules, which match actual usage patterns, and are associated to external factors like temperature, humidity, user presence, etc. The goal of our work, on the other hand, is to create personalized suggestions that are tailored to a household's energy consumption and usage of appliances and assist in achieving energy footprint reduction objectives by reducing excessive energy use.

Table 1 summarizes the main works on energy consumption profiling and their main features. Our proposed work differs from them in that it combines a novel data engineering technique to disaggregate energy-usage data from a single meter with pattern extraction algorithms and a recommendation engine that takes advantage of the extracted knowledge to promote energy savings.

**Table 1:** Pattern extraction methods from energy usage data.

**Method**	**Item-sets**	**Pattern type**	**References**
Association rule mining, Incremental Frequent Pattern extraction	Appliances that are on or off at the same timeslot	Inter-appliance relationships	Singh and Yassine, 2019
Sequential rule mining	Appliances that are used together	Appliance combos	Ong et al., 2013
Percentage change in consumption, k-Nearest Neighbor days, Histogram buckets	Appliance usage over time	Abnormal usage of appliances	Sial et al., 2021 Sial et al., 2018
Manual classification	Power consumption per time slot	Peak energy usage slots Location groups based on energy usage	Sial et al., 2014
Association rule mining	Appliances that are turned on or off per timeslot	Associations of on or off events with timeslots	Sardianos et al., 2019 Alsalemi et al., 2020

### 2.3. NILM and energy saving recommendations

Adomavicius and Tuzhilin (2011), discuss the importance of “situated actions” in recommender systems. The absence of information about the overall situation, including the user and environment state, the user habits etc., restricts the ability to provide more useful and smarter recommendations. Various contextual factors such as time and location of the performed action, the purpose of the action, etc. need to be used to make powerful recommendation systems.

Several studies have looked at the challenge of reducing energy use from the perspective of environmental psychology. The work of Abrahamse et al. (2005) is a survey of interventions targeted at household energy saving, and concludes that users either want to change or are motivated by a reward. Knijnenburg et al. (2014) conducted an experience research on the elicitation of users' preferences and found that the energy savings of a user depends on his/her awareness and knowledge of the operation of the system, and that this can be addressed by a simplified user interface. Starke et al. (2017) came to a similar conclusion, finding that picking personalized recommendations that are simple to implement leads to more energy-efficient choices and user satisfaction. In a later study, they discovered that users who seek advice to reduce energy consumption have more trust in recommender systems and their output. As a result, giving individuals a choice of recommended actions, can improve their trust to the recommender system (Starke, 2019). This field of research also adds the dimension of psychology to recommender systems, and examines how they can be more easily adopted by end users regardless of how the recommendations are created. Such discoveries influenced the design of our system's user interface.

There exist several research projects that are motivated by these findings on how psychology can be an important factor in energy-efficient choices and perform NILM and/or some sort of appliance usage pattern extraction in order to promote better energy consumption profiles and trigger behavior change. Garcia-Garcia et al. (2017) employ an open IoT data management platform and a gamified approach to help users adopt energy saving habits. ChArGED (Dimitriou et al., 2018) is a similar framework that combines IoT for sensing, NILM and pattern extraction *via* analytics, as well as serious games for motivating users to reduce power waste in public buildings. A similar framework is proposed in the BENEFFICE project (Garbi et al., 2019) where the authors propose to leverage IoT devices to capture appliance usage patterns and recommendations, incentives and challenges to gradually shape a better energy consumer profile. Most recently, the ENTROPY project (Ramallo-González et al., 2022) presented a series of educational interventions that promote energy saving in a timed and personalized manner. Going one step beyond, the EM3 project (Alsalemi et al., 2019) builds on the habitual behavioral change and combines context-aware recommender systems and IoT in order to maximize the probability of recommendation acceptance and thus the impact of the behavioral change policy. The results reported from a pilot study of EM3 on a office building setup demonstrate the power of properly timed recommendations in saving energy by promoting better habits (Sardianos et al., 2020).

The proposed work builds on the same motivation and concepts as the aforementioned systems. Our objective is to encourage and improve environment-friendly energy consumption actions by interacting with the user. The proposed framework supports non-intrusive load monitoring and analytics, and is powered by usage pattern extraction algorithms to generate real-time recommendations *via* a user-friendly application. As far as load monitoring and energy disagreggation is concerned, we treat NILM as a classification task, with the goal of detecting (predicting) on/off events for each appliance in different time segments (where “off” is not necessarily the same as 0 energy consumption, depending on the appliance). While the majority of previous work uses data analytics to create energy usage patterns, in this work we use data mining, and in particular explore how association rules analysis and sequential pattern mining can be used as the back-end of a recommender system. Compared with the most relevant work to ours (Alsalemi et al., 2019) that also employs similar algorithms, the main difference is that we extract a usage profile per household and we associate appliances that are frequently used together at each time segment. This coarse grain grouping of measurements in 30 min slots allows to extract more complex associations (larger itemsets), which is not feasible when we examine each appliance separately. The resulting usage patterns are directly connected with time, and thus can be used to trigger on/off recommendations. Through the proof-of-concept application, the user receives recommendations and is able to control (i.e., turn on and off) the household appliances.

### 2.4. Datasets

Energy consumption datasets are required to perform analysis, modeling and evaluation of energy usage patterns to recommend energy-efficient actions. A wide variety of such data sets are available, collected under different research studies but probably with the same goal of understanding user energy consumption needs and appliance level energy consumption patterns.

The individual household electric power consumption dataset (Hebrail and Berard, 2010), or UCI for short, can be found in the machine learning dataset repository provided online by the University of California, Irvine. The dataset consists of time-series based energy consumption data. Columns in the dataset include household global minute-averaged active and reactive powers, minute-averaged voltage, household global minute-averaged current intensity and minute based meter readings for three categories of appliances—kitchen, laundry, and central AC. The dataset consists of more than 2 million records collected from a house in Sceaux, France over a period spanning from December 2006 to November 2010. Sardianos et al. (2019) have used this dataset to train and evaluate their model for finding user energy consumption patterns and recommend energy-efficient actions.

Gao et al. (2014) introduce a public dataset named Plug-Level Appliance Application Dataset (PLAID) which consists of current and voltage measurements for over 200 appliance instances of 11 unique appliances. The dataset can be used to identify the different household appliances based on their load measurements. Due to its vast appliance data, the dataset proves to be very useful in varied applications such as identifying any appliance based on the calculated voltage-current values. But it proves to be insufficient in our project which aims to generate recommendations for optimum energy usage. The data values do not take into consideration the time of the day which is an important entity required for the analysis of energy usage. Moreover, it fails to gather and sort the appliance data per household for a particular user. This proves to be a major drawback of PLAID to be of use in the current application.

Residential Energy Disaggregation Dataset (REDD) (Kolter and Johnson, 2011) is another dataset used by researchers to develop algorithms aimed at separating an aggregate energy signal into its component level contributions and finding other important analytics. The data was collected from around 40 homes in Boston and San Francisco metropolitan areas. Monitoring devices were installed at the homes over a period of 18 months to collect the data. The whole-home aggregate electricity signals are recorded only at those times when the voltage and current at high frequencies show a change in the waveforms, i.e., when a device in the home is turned on or off. However, per-circuit electrical power is monitored every 3 s. Per-plug electrical power consumption is also monitored at a varied frequency for different houses, ranging from once per second to once per minute. The per-circuit and per-plug signal data provide the ground truth to the energy consumption patterns in the house.

REFIT (Personalized Retrofit Decision Support Tools For UK Homes Using Smart Home Technology) (Murray et al., 2017) is a dataset that contains electrical load measurements from 20 houses that were monitored for 2 years, while the occupants were performing their daily routines. The data has been employed in research works that propose and validate NILM methods, extraction of energy usage patterns etc.

Researchers at the Department of Computing at Imperial College London recorded electricity consumption of five households over periods of 3 months to 3 years (Kelly and Knottenbelt, 2015). The subjects were MSc or PhD students at the Imperial College. They recorded both the mains and individual appliance level power demand every 6 s. In each house they recorded both whole house mains power demand as well as individual appliance level power demand. Active power drawn by each appliance and whole-house was recorded every 6 s, resulting in 14 Gb of data. In order to record appliance level power demand, researchers installed individual appliance monitors (IAMs) between each appliance and its wall socket. UK-DALE dataset stands out because of simultaneously recording the power drawn by most of the individual appliances as well as overall power consumed by the house. This allows us to evaluate energy disaggregation approaches in a much more meaningful way. Also, having actual appliance-level data makes it easier to analyse appliance-appliance and appliance-mains associations which we will discuss in detail.

Our study employs the UK-DALE dataset, both for the experimental evaluation and the POC implementation. The main reason for this is that compared to other datasets used in the related literature, such as PLAID, REDD, UCI and more listed in Sun et al. (2019), this is the only dataset which simultaneously records the power drawn by most of the individual appliances as well as overall power consumed by the house in a very fine level of detail. This allows to evaluate different energy disaggregation approaches (available in the NILMTK library; Batra et al., 2014), and extract appliance-appliance and appliance-mains associations. The datasets and their main features are summarized in Table 2. A detailed overview of the UK-DALE dataset is included in Table 3. Since data collection is not consistent across all houses, we will be treating each house as a separate case.

**Table 2:** Popular NILM and energy usage datasets.

**Shortname**	**Different appliances**	**No. of households**	**Duration**	**References**
UCI	9	1 × 3 rooms	47 months	Hebrail and Berard, 2010
PLAID	17	65	6 months	Gao et al., 2014
REDD	16	40	18 months	Kolter and Johnson, 2011
REFIT	9	20	24 months	Murray et al., 2017
UK-DALE	>10	5	3–36 months	Kelly and Knottenbelt, 2015

**Table 3:** The main features of the UK-DALE dataset.

**House**	**1**	**2**	**3**	**4**	**5**
Building type	End of terrace	End of terrace		Mid-terrace	Flat
Year of construction	1905	1900		1935	2009
Energy improvements	Solar thermal, loft insulation, Solid wall insulation, double glazing	cavity wall insulation, double glazing		loft insulation, double glazing	
No. of occupants	4	2		2	2
Total no. of meters	54	20	5	6	26
Date if first measurement	2012-11-09	2013-02-17	2013-02-27	2013-03-09	2014-06-29
Date if first measurement	2015-01-05	2013-10-10	2013-04-08	2013-10-01	2014-11-13
Total duration (days)	786	234	39	205	137
Avg. mains energy consumption per day (apparent kVAh)	8.90	8.00	12.35	10.24	17.56

## 3. Non-intrusive load monitoring techniques

In the absence of smart meters, the first core module of the process is the energy disaggregation, also known as non-intrusive load monitoring (NILM). This module encompasses extracting the available appliance's individual power consumption and state of operation from the main meter reading. As we discussed previously, NILM is a more preferred solution to load monitoring as it allows to obtain appliance level data without intrusion from third party devices such as smartplugs, sensors etc., which preserves user's privacy. Also, installing smart plugs for getting appliance data is an arduous task and often impractical and not cost-effective.

The fundamental idea behind the NILM technique is to recognize step changes in power consumption and, based on the size of the step, to identify the appliance that caused the change. It also helps in mapping the energy signature or footprint of an appliance. The availability of ground truth (i.e., the individual appliances' consumption) in the UK-DALE dataset, allowed us to explore various machine learning algorithms. In particular, we consider a clustering technique proposed by one of our team members (Sardianos et al., 2019), and three state-of-the-art disaggregation techniques that are included in the NILMTK library (Batra et al., 2014, 2019).

**Figure 2:**
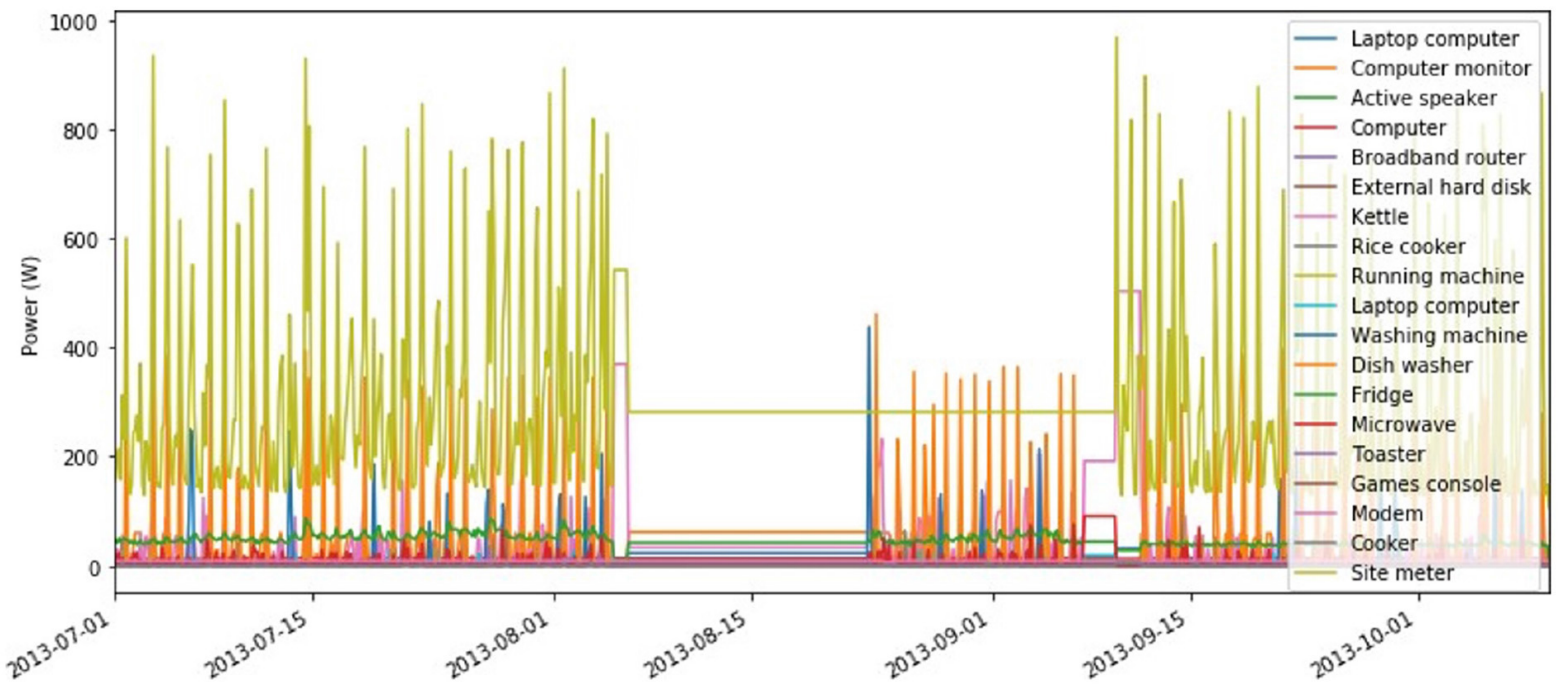
House 2 appliance's and mains power consumption.

As discussed in previous sections, the UK-DALE dataset that is chosen for this project ranges over five different houses containing mains power as well as each appliance's power consumption data over varying time periods. The appliance data is recorded in approximately a 6 s period. However, the raw data had irregular time intervals. Therefore, the data had to be re-sampled over a period of 120 s before the data was used for training. A visual representation of a subset of the data (House 2 appliances) over a 3-month period is shown in Figure 2. As one can see on the legend of the plot, the house comprises a wide range of appliances from computers and peripherals to white appliances. However, it contains no heating, ventilation and air conditioning (HVAC) devices.

Hart's 1985 algorithm was the first algorithm designed for NILM (Hart, 1985). This unsupervised algorithm monitors voltage and current changes in the main meter and does a cluster analysis to identify various appliances switching operating states. Clusters are formed using the step change in voltage. Each cluster is then considered as one device and then using the ground truth, the algorithm finds the best matched appliance for each cluster. The voltage change plays a crucial part in identifying different appliances' states. Hence, devices with significant effect on power changes such as refrigerators can easily be identified, whereas all other appliances are hardly distinguished.

Combinatorial optimization (CO) during training forms clusters from each device's power consumption to have a set of power states. Based on these clusters, the algorithm designs a 2D matrix, where each column represents an appliance and each row represents a combination of appliances and their aggregated power consumption. At disaggregation, the algorithm just finds the aggregated sum nearest to the main meter reading, which associates a combination of appliances turned on to a particular reading. In the NILMTK implementation of this algorithm that we used in our framework (Batra et al., 2019), only the submeters' data is used for training with this supervised algorithm. Due to irregularities in intervals between consecutive readings, the model is trained data resampled to 2 min intervals. Once the model is trained, it can predict on the test data, which are collected from the main meter's readings. At the same time, the model keeps building ground truth data collected from the submeters of test data for comparison.

The Factorial Hidden Markov Model (FHMM), during training, fits a Hidden Markov Model (HMM) to every appliance using disaggregated ground truth data and then integrates all the HMMs. While predicting, the algorithm runs the Viterbi algorithm to predict the best sequence of appliances that are turned on. Similar to CO, in the NILMTK implementation (Batra et al., 2019) this algorithm also uses submeters' data for training the model with data resampled to user's preferred interval. The difference while training the model with FHMM compared to CO is that FHMM uses more memory than CO. Due to this the algorithm complexity increases with the number of appliances and the algorithm is more memory demanding than CO. To tackle this problem, we trained the model by dividing the appliances in batches and subsequently combining the results to get all appliances' data.

Finally, we explored the clustering technique used by Sardianos et al. (2019) to disaggregate datasets and get the appliance level switch on/off events. For a number *N* of devices in a household, we form *k* = 2^*N*^ clusters by applying the K-Means clustering algorithm. We do this twice—for both the positive and the negative energy consumption change values—to get the individual appliance switch on and off events respectively. The resulting clusters (which correspond to composite on/off events) are sorted using their centroids, in increasing order of energy demand, to get a mapping between the cluster ids and the actions. Next, we find the status of individual appliances at every timestamp in the dataset by comparing their previous energy reading with the current change. This lets us derive what devices were switched on or off at what time of the day. Here we can clean up the dataset by discarding certain rows that show no change in the status of any appliances corresponding to their previous energy readings. The resultant dataset is disaggregated with information about device switch on and off actions along with date and time of the day. This data set can be further used to extract user habits by applying rule mining algorithms, as discussed in the future sections. The drawback of this approach is that as the number of appliances *N* increases, the number of clusters and combinations increase exponentially (2*2^*N*^). Hence, the disaggregation approach becomes more cumbersome as the number of appliances in a house increases. For instance, in the case of houses 1, 2, and 5 from the UK-DALE dataset that have more than 18 appliances each, this approach would require the creation of more than 500 thousand clusters. We therefore did not explore it any further.

## 4. Appliance usage recommendations

The type of data that is fed to a recommender system is one of the main issues to consider in the recommendation generation process. Power consumption readings are the main input of the system, and this must be somehow converted to suggestions for which appliances to turn on or off at specific times of the day. Data pre-processing is the first step in this process and this has to be followed by the right pattern mining method that will provide a number of comprehensive appliance usage patterns. The last step is to use these patterns in order to generate recommendations. In this section, we present our methodology in detail and illustrate the results of our analysis. For simplification, we assume that a NILM or IAM technique is employed and consumption data are already disaggregated when fed to the recommender system.

### 4.1. Data preprocessing

The extraction of appliance usage patterns, from the disaggregated consumption measurements for multiple appliances, assumes that we are able to identify when individual appliances or appliance combos are turned on or off. This is important since we decided to resample the initial readings in 30 min time intervals (which we call *day segments*), taking the average consumption of all the readings (per appliance) during that interval. This yields 48 values per device each day compared to the initial 1,440 min readings per day, and has boosted the system's scalability. Assuming that users do turn on/off multiple appliances at the same time, the derived patterns are richer and increase the flexibility of recommendations.

This pattern extraction task is not simple because many appliances consume power when they are plugged in, even when they are in stand-by mode. Therefore, we must determine the range of power consumption values for each appliance that denotes its actual usage.

In our initial effort to address this, we employed the following equation to derive a mean-based threshold value that would classify a particular reading into an on or off state: $threshold=\frac{maxReading-minReading}{meanReading}$, where *maxReading, minReading*, and *meanReading* are the maximum, minimum, and average readings respectively of the appliance under consideration for the period of reference. For that period of the day, the appliance is recorded as *on* if the current reading exceeds the threshold value; otherwise, it is marked as *off*. For appliances that are not used frequently, and for which we had few data many false events can be detected. Since slight increases in energy usage in these circumstances are ignored, they were identified as *always off*.

A second method, which was based on K-Means clustering (with *K* = 2), has been developed to increase classification accuracy. More specifically, each appliance's readings were split into two clusters using K-Means. The *off* label was assigned to the cluster with lower value readings, and the *on* label to the cluster with higher value readings. As the less-frequently used appliances were being accurately categorized, employing K-Means as opposed to the threshold for determining appliance on/off status proved to be a preferable technique.

### 4.2. Household profile

We can create an appliance consumption profile for each user/household based on the appliance on/off events that we collect every day. The profile is extracted for each household and contains all the appliance power consumption periods.

We designed the household profile per day with day segments as keys, and the respective list of appliances switched on at each segment as values. Done for the entire time period, this results in a nested list with as many records as the days in the training set. The first element of each nested list representing a day is again the day segment followed by the appliances switched on at that time in each household. This is done to make sure that day segment is considered as one of the frequent items and will be included in the patterns identified by the mining algorithm. These sequences of appliances switched on at a given time segment are used as an input to a pattern mining algorithm. In essence, we can process the lists of appliances utilized in the same day segment across all days in order to detect recurring usage trends.

Our proof-of-concept implementation employs household profiles from the complete dataset (i.e., over the entire time period covered by it). Note that the framework can be easily updated to include seasonality. In essence, instead of deriving a single household profile, we can use the power demand readings for each season to derive the household's season profile, and this can be used to produce suggestions for specific months or seasons. Similarly, we can split the dataset using other types of filters, such as weekdays vs. weekends, etc. To find recurring patterns in the home power usage profile, we constructed and assessed a number of association rule and sequential pattern mining methods.

### 4.3. Appliance usage pattern mining

In order to extract patterns of appliance usage from the household profiles, our system design enables a number of association rules and sequential pattern mining methods. *Apriori*, FPGrowth (both as implemented in Pedregosa et al., 2011), and TRuleGrowth (Fournier-Viger et al., 2012b) (as implemented in Viger, 2008), are the three most effective algorithms in terms of scalability and compactness of model without sacrificing coverage. Appliance sets that are frequently used together in the same day segment are discovered by *Apriori* and FPGrowth, and are stored in a dictionary. Since there is no dependency between appliances used at different day segments, this step can be easily parallelized for the different segments. The next step is to extract rules from the frequent appliance sets of each day segment and use these rules as the user's behavior concerning the usage of appliances at the specific segment.

**Figure 3:**
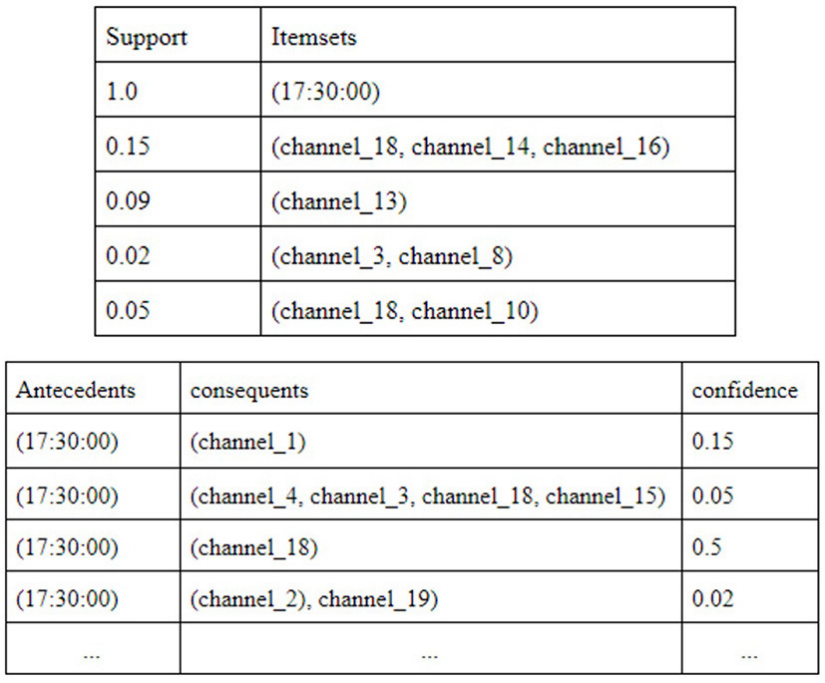
Frequent itemsets and association rules for House 2.

Figure 3 shows a snapshot of the generated rules for House 2. House 2 has 18 different appliances and we found that minimum support of 0.02 generates frequent itemsets (candidate set) which include 17 out of the 18 appliances in the house, the remaining appliance being Playstation console. During the data exploration phase we found that the Playstation console is the least used appliance in House 2. Frequent itemsets generated with minimum support of 0.01 also did not include Playstation in the frequent itemsets and increasing support to 0.03 resulted in losing more appliances from the frequent itemset pool. So we decided to choose 0.02 as the minimum support value to generate frequent itemsets.

Additionally, we assessed a number of sequential pattern mining techniques from the SPMF library (Viger, 2008), we found appliance-time dependencies and calculated the likelihood that an appliance will be on at a specific time. The execution time, the memory requirements of each algorithm, and the number of generated rules have been evaluated for all methods. CMRules (Fournier-Viger et al., 2012a) is an algorithm that finds sequential patterns using the similarity of sub-sequences among longer sequences. RuleGrowth (Fournier-Viger et al., 2012b) employs a recursive technique for expanding smaller sequential patterns. TRuleGrowth (Fournier-Viger et al., 2012b) can generate rules from long sequences since it employs a maximum sliding window. ERMiner (Fournier-Viger et al., 2014) groups rules with the same prefix (or suffix) into equivalence classes and a filters out sequences using a sparse count matrix. Finally, CMDeo is based on an older algorithm for pattern extraction from single sequences (Deogun and Jiang, 2005) and has been adapted to handle multiple sequences. From all these algorithms we choose TRuleGrowth for extracting time-related patterns from our dataset.

**Figure 4:**
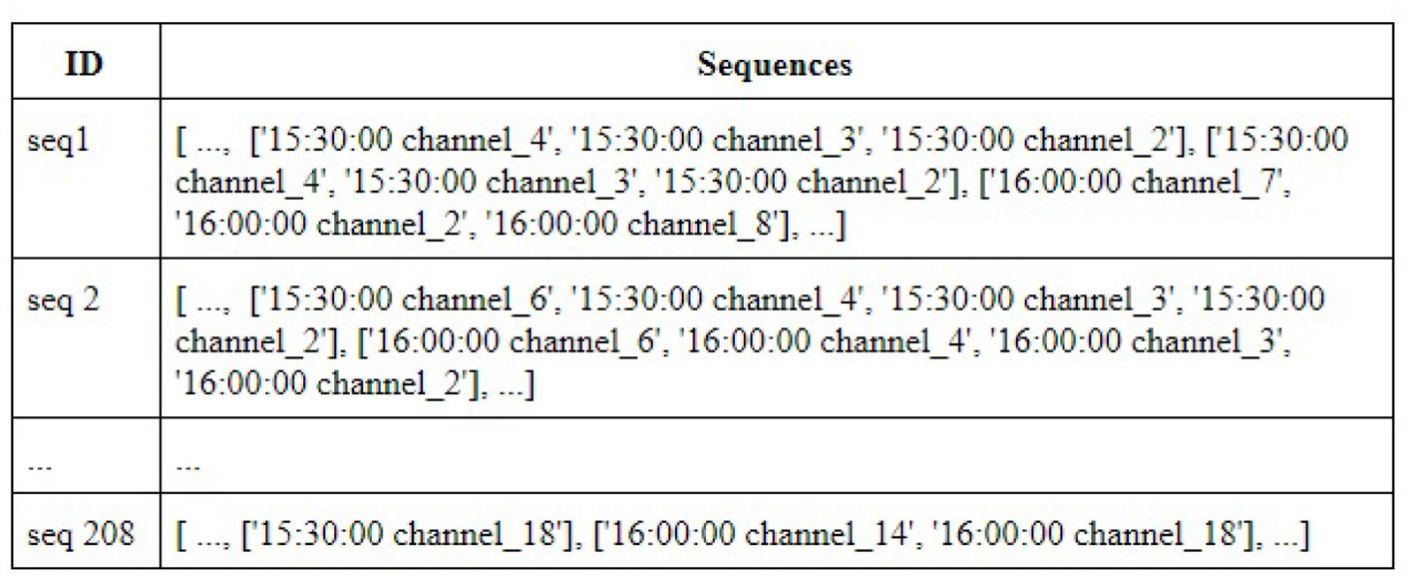
Sequences for House 2.

In particular, for the same input, CMRules and ERMiner both faced memory scalability issues and crashed before completion or took hours to complete. While faster than these two, and due to its recursiveness, RuleGrowth proved costly for datasets with long sequences like ours. In addition, the processing generated about 26.3 GB of data, almost 9 times the output size of TRuleGrowth. The left and right expansion process of CMDeo result in support being the only metric to use to search for patterns in the sequences, as confidence does not remain stable. In addition, CMDEo finds only subsets of all valid rules. As a matter of fact, the algorithm generated only 585 rules. In the contrary, TRuleGrowth generated 44,155 rules in 12 s, and generated a file size of around 163 MB. This is consistent with the results presented in Fournier-Viger et al. (2012b), which show that TRuleGrowth is memory-efficient and can quickly provide rules. Figure 4 depicts a snapshot of the TRuleGrowth-produced sequences for House 2.

From the resulting rule set, we only keep the rules that have a single time segment and an appliance. This allows to create a collection of rules that have the following structure < *time*_*segment* → *appliance*_*channel* >. The rules, along with their support and confidence scores, are fed to the recommendations module.

Using this subset of generated sequences, we can generate a map of appliance names and their active time slots. From this data, we get the knowledge about when a user uses a particular appliance in the whole day. This can be directly related to the user habits as we can successfully make the connection between the appliance state and the user activity.

### 4.4. Recommendations

**Figure 5:**
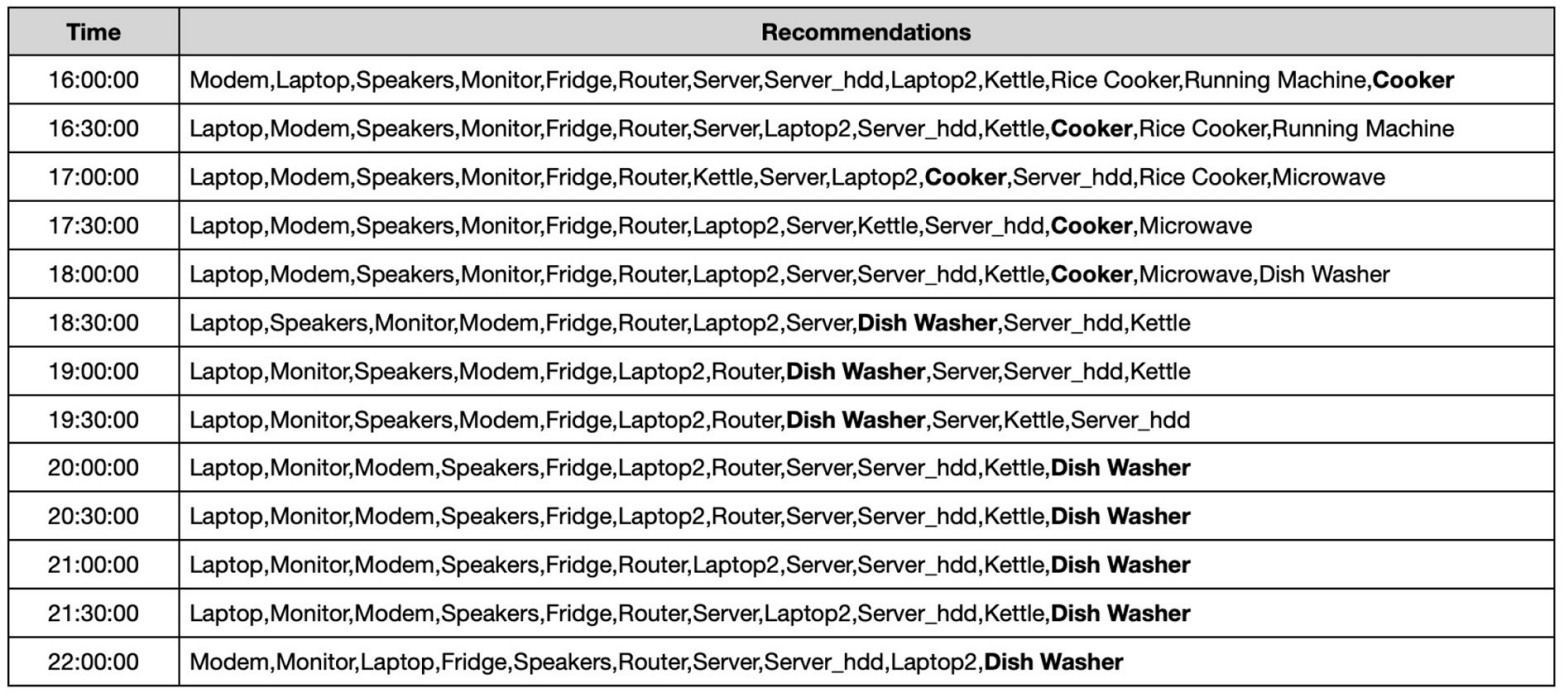
Recommendations for House 2 (between 16:00 and 22:00).

This is the real-time module of the proposed framework. For a given time slot the system extracts the rules for this *time_segment* and sorts them by *confidence*. The system then selects the top-k rules (where k is also defined in the UI) that match the appliances used at that moment by the user, and generates the respective recommendations. In the end, we get a list which consists of the appliances to be on at a particular time of the day. The list consists of 48 instances corresponding to the 30 min time intervals. The frequently utilized appliances that are turned on in different time segments are displayed in Figure 5. These itemsets have been used to generate rules and recommendations for House 2.

**Figure 6:**
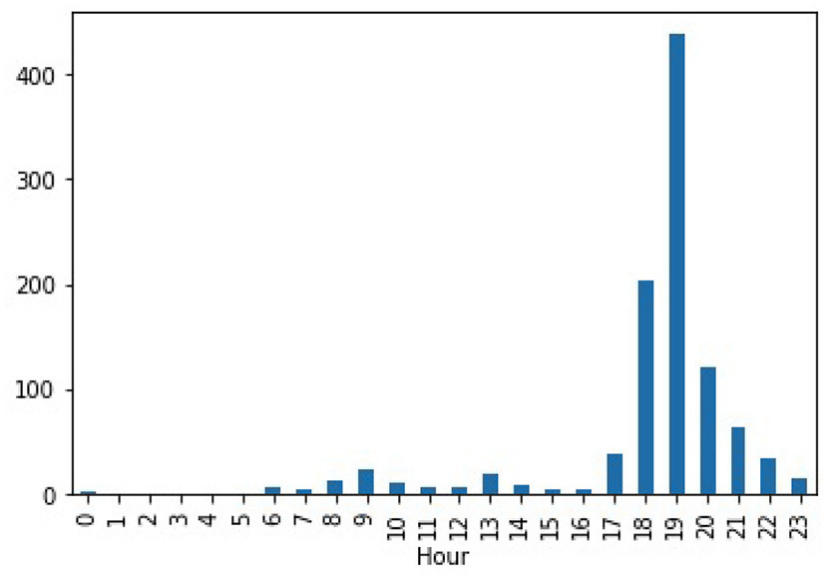
Avg. power/hour for Dishwasher in House 2.

We empirically discovered that the recommendations were consistent with the user's regular activities at home and the actual power consumption of the appliances, as seen in both the raw data and the previously identified usage patterns. Figure 6 shows the actual appliance level power demand data from the UK-DALE dataset for House 2 Dishwasher aggregated at the hour level. We notice that the dishwasher uses more energy on average between the hours of 6 and 10 p.m., which is when the system suggests turning it on. Other appliances also follow a similar trend.

The evaluation of the recommendations' performance is based on these findings. We assess the effectiveness of the suggestions using precision, recall, and their harmonic mean, the F1-score, in Section 6. We also employ t-SNE to form appliance clusters based on their usage and plot them, as another indirect evaluation of the identified patterns.

## 5. Proof-of-concept

As a proof-of-concept (POC), we developed an application with a web dashboard that allows the user to monitor their house and appliance energy consumption, and a customizable panel where the recommendations appear (the application is built using the ReactJS framework and can be deployed on any server).

**Figure 7:**
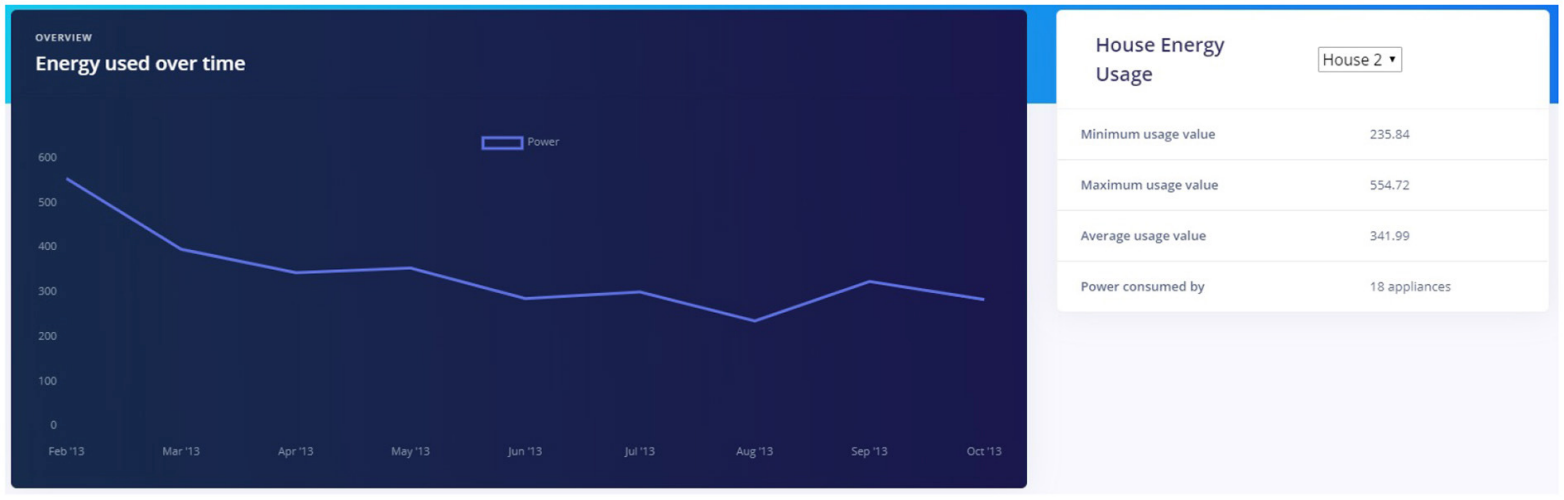
Energy metrics for House 2.

The Dashboard page presents the user with a summary of home aggregate power usage and appliance wise power usage using graphs. The house aggregate power usage data is sampled into month intervals and the section highlights minimum, maximum, and average power usage over the available time period. For our POC, we have used the historic data as found in the UK-DALE dataset. A screenshot for House 2 is shown in Figure 7.

**Figure 8:**
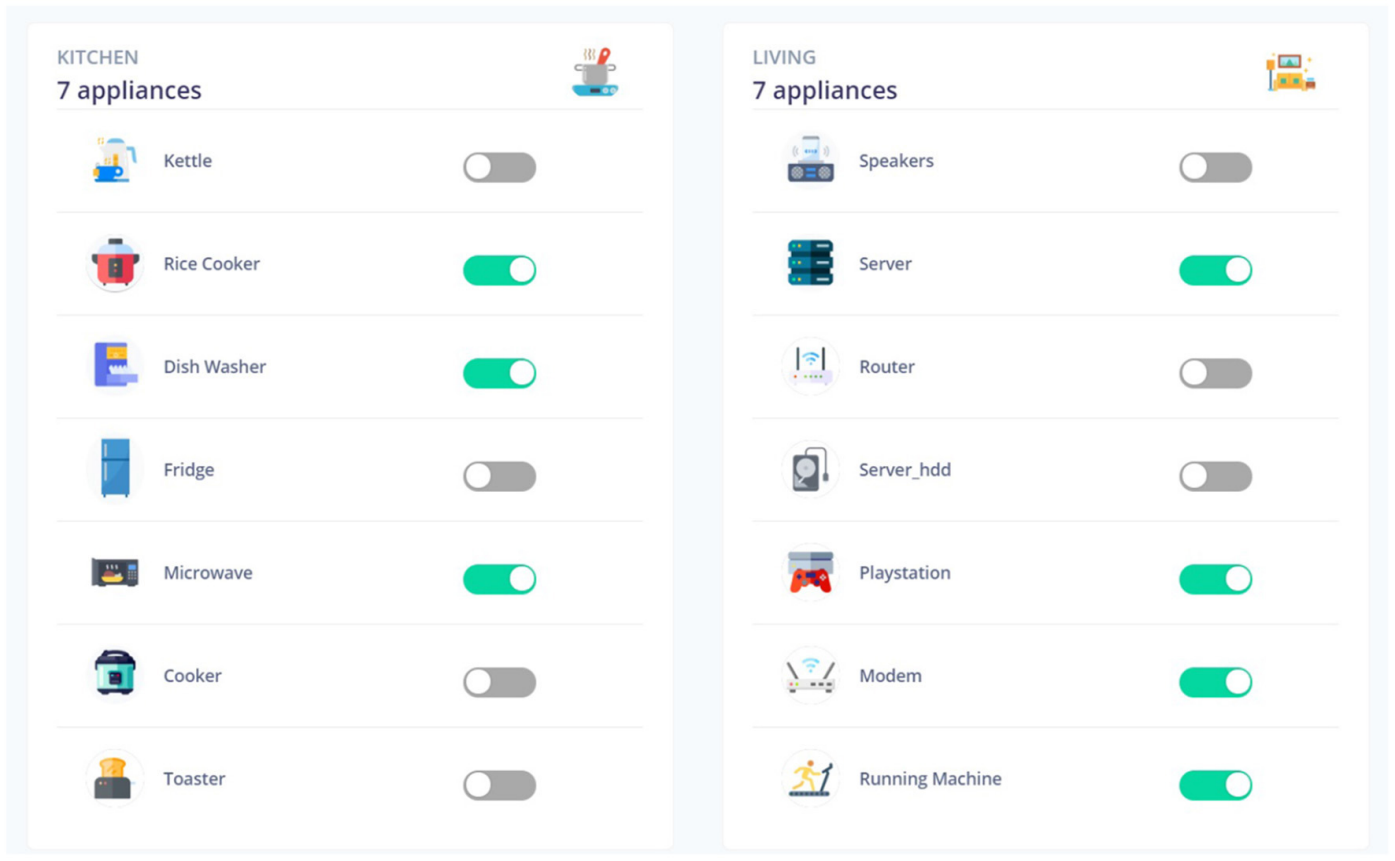
Appliance control for Kitchen and Living room.

**Figure 9:**
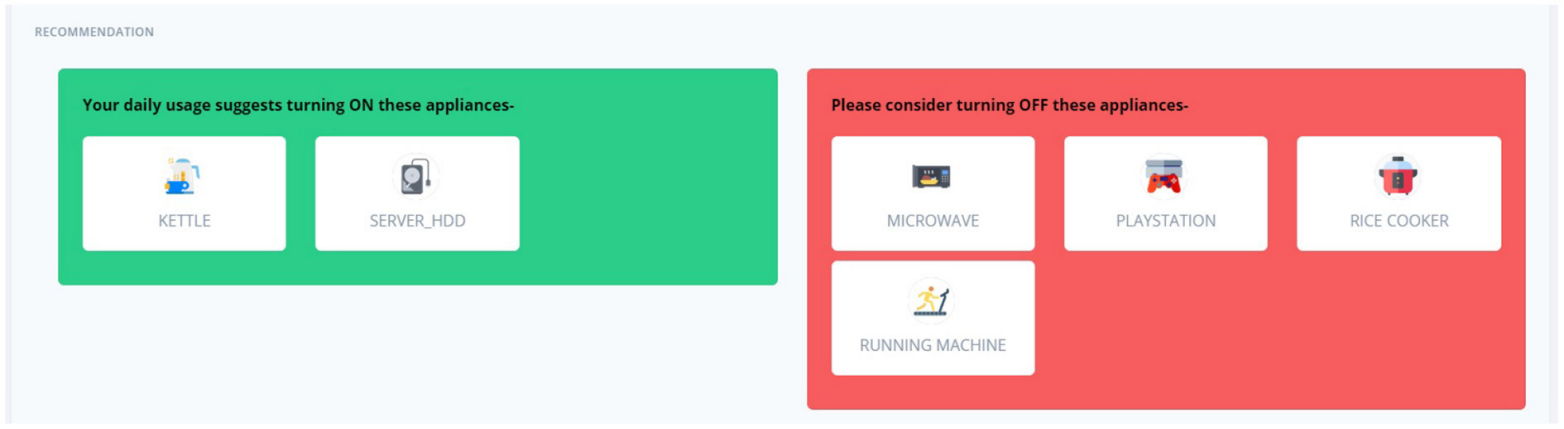
Energy saving recommendations.

Through the UI, the user can view all the appliances in the house grouped by room, as shown in Figure 8 for the Kitchen and Living room of House 2. To simulate a real-life scenario, where the application monitors the power demand of the household and identifies which appliances are currently on, we can turn any device on/off and pick a time of day. Based on this input, and the recommendation list generated by our back-end system described in Section 4, the system suggests which appliances need to be turned on or off with respect to the current state of the house, as shown in Figure 9. Through the interactive UI the users can monitor the power consumption of their households, can get recommendations for better appliance usage and respond to them by using the appliance on the right time or switching them off when they do not need them.

We must keep in mind that the system also suggests to turn appliances on. This may act as a notification system in a variety of situations, such as unexpected power outages while a user is gone (informing them to turn on the refrigerator again) or energy-saving practices (user is reminded to turn on the dishwasher during nighttime when the charge is lower).

Additionally, the user can filter out appliances and mute recommendations for them (e.g., appliances that are always on, like a router or a fridge) or set specific energy objectives for the consumption of an appliance. The implemented prototype also offers the option to use voice commands in order to increase accessibility.

## 6. Experimental evaluation

We evaluate our framework both empirically and quantitatively, using the UK-DALE dataset (Kelly and Knottenbelt, 2015), as previously discussed. We first perform a benchmark for the most popular NILM algorithms as implemented in NILMTK over UK-DALE's Houses 2–5 data. Here we present the results of the two best-performing algorithms, namely Factorial Hidden Markov Model (FHMM) and Combinatorial Optimization (CO), for each house and for each appliance type across all houses. The results vary between appliances and houses. However, in general FHMM outperforms CO in terms of accurately predicting (as measured by macro-averaged Accuracy, Precision, Recall, and F1-score) the appliance's usage. More specifically FHMM has better precision than CO in detecting when a device is switched on or off based on the aggregate measurement and a slightly better F1 score. Note that, while in this work we focused on NILMTK implementations, our framework is modular and can accommodate newer NILM algorithms, as those discussed in Section 2. We then employ the t-SNE algorithm to visualize the discovered appliance/time associations and sequential patterns. Upon visual inspection we verify that some appliances that are expected to be used together are indeed assigned to the same cluster. This validates our choice of algorithm for appliance usage pattern mining.

Finally, we focus our quantitative evaluation to the core module of the proposed framework that is the recommender system. We use four houses that demonstrate different characteristics in terms of days of recordings, number of appliances, total and average daily consumption. We generate recommendations and observe that overall the algorithm accurately predicts appliance usage achieving high recall (i.e., the system correctly predicts appliances that were actually on). In addition, and most importantly, we estimate how much energy could be saved if the users followed the recommendations and turned appliances off as prompted. We show that these savings range from 2 to 17%. While our framework differs from other research projects with similar objective in terms of setup and parameters (source of readings, input, type of analysis, and form of recommendation, dataset) making direct comparisons difficult, we should note that our findings are in accordance with other recent studies, such as Ramallo-González et al. (2022) that measured how much energy can be saved if users follow prompts or recommendations for energy preservation, or Sardianos et al. (2020) that reported savings from the application of their recommender system on a office building setup.

### 6.1. Energy disaggregation benchmarking

One of the reasons why we selected the UK-DALE dataset is that it includes both the total energy consumption, as well as the disaggregated appliance-level energy consumption, which can be used as ground truth. This ground truth can be used to evaluate the various disaggregation algorithms. For the evaluation we use Houses 2 and 5, which are big houses with many appliances and Houses 3 and 4 that contain a small number of appliances each (four and five appliances, respectively). We omit House 1 because it has many appliance channels that contain composite measurements even in the disaggregated data (e.g., combos of living room lamp and tv, kitchen phone, and stereo, etc.), which makes the task of finding the device signature and compare across houses much harder. The output of the disaggregation algorithms assigns a specific energy consumption ${\hat{x}}_{i}$ to each appliance for each time period *i*. As previously discussed, we focus on two algorithms of the NILMTK library (Batra et al., 2019), namely Combinatorial Optimization (CO) and Factorial Hidden Markov Model (FHMM). We did not further consider the other two options as they each had shortcomings, as previously discussed.

We then compare the two algorithms in terms of predicting the on/off state of each appliance. Please remember that some appliances record energy even in their off (idle) state, if they are plugged in. Therefore, for the ground truth data, we first calculated the mean and labeled as “on” all values that were above that threshold. The disaggregation algorithms generate output in a different way: for each time period, the appliance is either labeled as having 0 energy consumption, or a value. We therefore labeled 0 as the “off” state, and any reading as the “on” state. We then measure the precision $\left(P=\frac{\left|correctly\text{\_}pred\text{\_}on\right|}{\left|pred\text{\_}on\right|}\right)$, recall $\left(R=\frac{\left|correctly\text{\_}pred\text{\_}on\right|}{\left|on\right|}\right)$, and F1-score for all appliances and all houses. As depicted in Tables 4, 5, the two algorithms perform differently depending on the appliance. Overall, we observe that FHMM outperforms CO for most of the appliances and overall in the houses we studied.

**Table 4:** NILM method performance in predicting the appliances' on/off status per house and overall.

	**FHMM**				**CO**			
**House**	**Accuracy**	**Precision**	**Recall**	**F1-score**	**Accuracy**	**Precision**	**Recall**	**F1-score**
2	0.31	0.92	0.23	0.33	0.67	0.40	0.30	0.31
3	0.21	0.83	0.07	0.13	0.61	0.64	0.10	0.17
4	0.34	0.85	0.24	0.34	0.53	0.68	0.24	0.32
5	0.39	0.97	0.37	0.50	0.57	0.48	0.41	0.43
Macro-average	0.31	0.89	0.23	0.32	0.60	0.55	0.26	0.31

**Table 5:** NILM method performance in predicting the on/off status per appliance.

	**FHMM**				**CO**			
**Appliance**	**Accuracy**	**Precision**	**Recall**	**F1-score**	**Accuracy**	**Precision**	**Recall**	**F1-score**
Laptop computer	0.36	0.81	0.27	0.39	0.48	0.57	0.23	0.31
Computer monitor	0.43	0.91	0.33	0.49	0.49	0.48	0.29	0.36
Active speaker	0.39	1.00	0.39	0.56	0.53	0.50	0.41	0.45
Computer	0.57	1.00	0.57	0.73	0.50	0.47	0.58	0.52
Broadband router	0.28	1.00	0.28	0.44	0.50	0.52	0.29	0.37
External hard disk	0.60	1.00	0.60	0.75	0.50	0.48	0.61	0.54
Kettle	0.11	1.00	0.11	0.17	0.76	0.55	0.22	0.27
Rice cooker	0.01	1.00	0.01	0.02	0.92	0.40	0.04	0.08
Running machine	0.08	1.00	0.08	0.15	0.77	0.20	0.09	0.12
Washing machine	0.24	0.78	0.01	0.02	0.91	0.45	0.07	0.09
Dish washer	0.03	1.00	0.03	0.06	0.98	0.38	0.65	0.48
Fridge	0.44	1.00	0.44	0.61	0.56	0.53	0.51	0.52
Microwave	0.29	0.71	0.02	0.03	0.95	0.23	0.11	0.15
Toaster	0.30	0.72	0.01	0.02	0.95	0.07	0.01	0.02
Games console	0.60	1.00	0.60	0.75	0.50	0.46	0.61	0.52
Modem	0.19	1.00	0.19	0.32	0.50	0.50	0.19	0.28
Cooker	0.46	0.59	0.02	0.05	0.47	0.49	0.02	0.04
Freezer	0.47	0.71	0.43	0.54	0.51	0.60	0.45	0.52
Boiler	0.38	1.00	0.38	0.55	0.41	0.77	0.37	0.50
Television	0.06	1.00	0.06	0.10	0.54	0.69	0.08	0.14
Electric space heater	0.47	0.56	0.01	0.03	0.89	0.61	0.20	0.31
Projector	0.12	1.00	0.12	0.22	0.13	0.64	0.06	0.10
Hair dryer	0.46	0.86	0.38	0.52	0.48	0.49	0.34	0.40
Network attached storage	0.43	0.91	0.33	0.49	0.49	0.48	0.29	0.36
Server computer	0.39	1.00	0.39	0.56	0.53	0.50	0.41	0.45
Electric oven	0.57	1.00	0.57	0.73	0.50	0.47	0.58	0.52
Electric stove	0.28	1.00	0.28	0.44	0.50	0.52	0.29	0.37
Vacuum cleaner	0.60	1.00	0.60	0.75	0.50	0.48	0.61	0.54
Audio amplifier	0.01	1.00	0.01	0.03	0.98	0.39	0.37	0.38

This is a good indicator for what would be the optimal solution for a dataset like UK-DALE. Given that this dataset already has disaggregated data, we performed our recommender system evaluation using the actual (ground truth) dataset. However, in the absence of such a dataset, we conclude that FHMM would be a good candidate to perform NILM.

### 6.2. Visualization of associations

**Figure 10:**
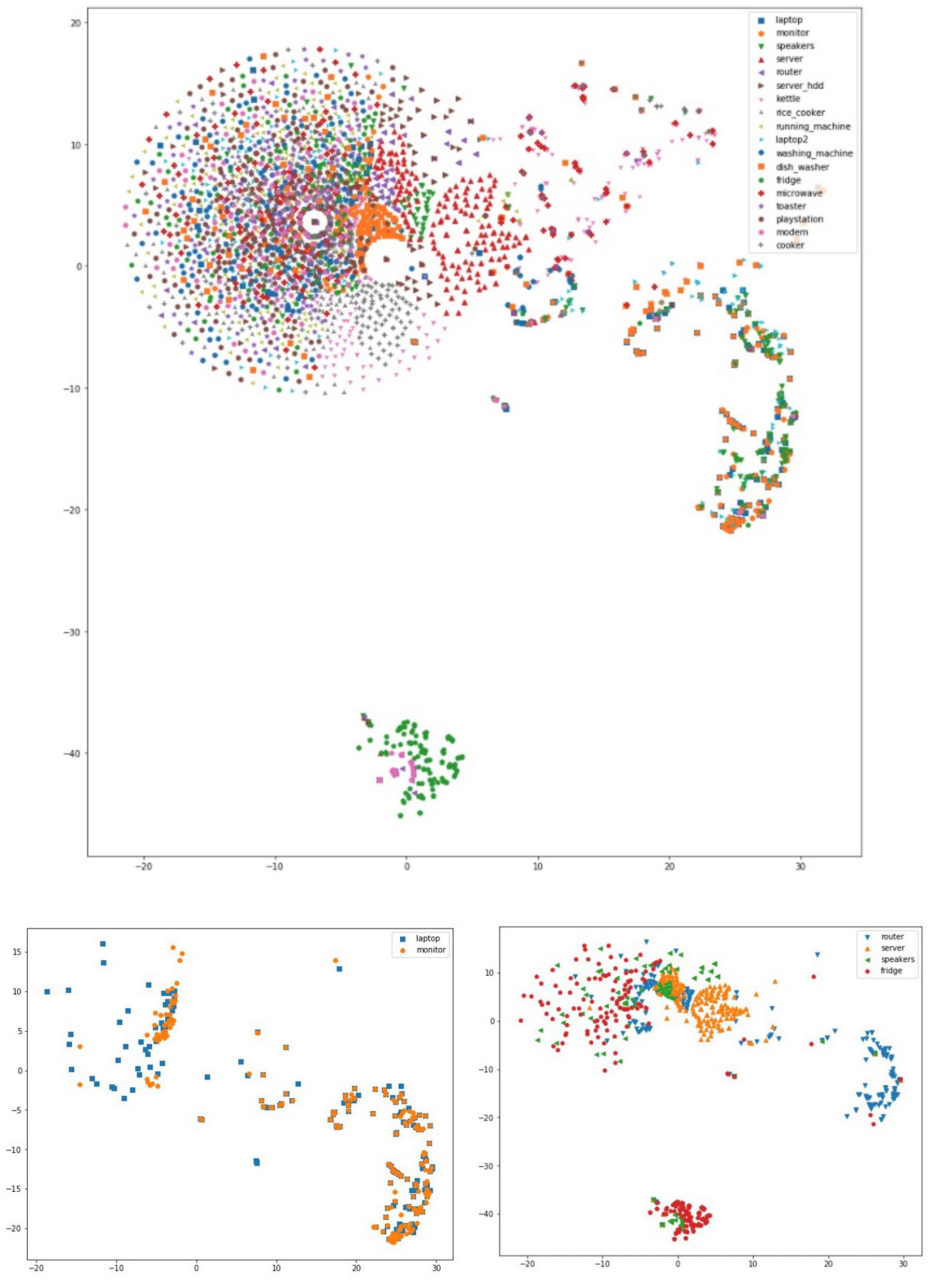
t-SNE clusters for House 2 appliances.

An indirect way to evaluate and verify that the discovered appliance/time associations and sequential patterns are valid is *via* clustering and visualization. For this reason, we employ the t-SNE algorithm (van der Maaten and Hinton, 2008) to visualize these appliances on a 2-D plane. To achieve this, we represent each appliance's usage throughout a day as a vector of on/off status. The vector consists of 48 elements, each representing a 30 min time interval starting with 00:00:00. Each appliance has one vector for each day of the date range in which the dataset is recorded. We create a vector list for each appliance in the house and input it to the t-SNE algorithm for plotting. Figure 10 shows all appliances in House 2, as well as some visualizations of subsets of the appliances. We observe several overlapping clusters, but within each cluster we find mini-clusters of appliances. One clear cluster is the fridge and modem, two appliances that are always on. For example, the router and speakers are clustered together tightly, and the same holds for the monitor and laptop that are clustered together as well, since the monitor will be used only with a laptop. We observed similar patterns for other appliance subsets.

### 6.3. Recommender system evaluation

The system evaluation has been performed using houses 2–5 from the UK-DALE dataset. These data span a wide range of appliances (from 5 to 25 per household) and monitoring periods (from 1 to 8 months). The data has been split using a 90:10 training-test ratio, using stratified sampling per household. The main properties of the dataset are shown in Table 6.

**Table 6:** UK-DALE dataset statistics.

**House**	**No. of appliances**	**No. of days in training/test set**	**Total consumption (Wh)**	**Avg. daily consumption (Wh)**
2	20	212/24	31,935.98	1,330.67
3	5	36/4	1,005.86	251.46
4	6	186/21	11,658.71	555.17
5	25	124/14	44,788.08	3,199.15

The test set's power consumption data was resampled and aggregated to determine which appliances are used the most frequently in each data segment. The precision *P* and recall *R* for up to 10 appliance recommendations were evaluated as follows: $P=\frac{\left|relevant\text{\_}rec\right|}{\left|all\text{\_}rec\right|}$, $R=\frac{\left|relevant\text{\_}rec\right|}{\left|ground\text{\_}truth\right|}$, where *relevant*_*rec* is the number of “true positives,” i.e., the appliances recommended “on” that were also on in the ground truth set. Table 7 shows the precision, recall, and F1-score averaged over all the time intervals on all days in the test set. Note that some readings in the dataset, such as these for House 3 spanned multiple appliances, resulting in a coarser prediction and recommendation. The algorithm accurately forecasts a household's energy habits in terms of appliance usage and achieves high recall at the expense of precision. It is important to note that varying *confidence* levels affect how the rules are covered. We tested various confidence thresholds. For higher thresholds, the number of appliances drops and thus less appliances are included in the recommendations. This is expected to yield worse results in terms of energy savings, and thus we report our results with the optimal threshold for the particular dataset. Please note that in this problem, the objective is to save energy while not compromising user satisfaction. This trade-off requires that we try to match the household's energy usage patterns and avoid recommending turning appliances off that are predicted to be on during that time. Thus, we opt for high recall, as previously mentioned. However, the UI provides the user the option to filter out appliances that they do not wish to get recommendations for (i.e., some sort of personalized post-pruning of the generated rules).

**Table 7:** Evaluation of recommendations.

**House**	**Precision**	**Recall**	**F1-score**
2	0.3	0.97	0.46
3	0.39	0.73	0.51
4	0.48	0.97	0.64
5	0.34	0.98	0.5

After that, we determined how much energy can be saved in each household if users follow the recommendation to switch off appliances. For each time period, we estimated the energy used by all the appliances that were on in the test set but were advised to be off by the system. This is energy that can be saved if recommendation are followed. The findings, which are described in Table 8, show that implementing such a recommender system could result in energy savings ranging from 2 to 17%. We found that the number of appliances has a negative correlation with energy savings, since the more *vital* (nearly always on) appliances a family has, the less is the impact on energy saving from turning off the remaining few appliances.

**Table 8:** UK-DALE energy saving statistics.

**House**	**Total**	**Avg. daily**	**Total energy**	**Avg. energy**	**%**
	**consumption (Wh)**	**consumption (Wh)**	**saved (Wh)**	**saved per day (Wh)**	**energy saved**
2	31,935.98	1,330.67	1,612.52	67.18	5.04
3	1,005.86	251.46	173.32	43.33	17.23
4	11,658.71	555.17	236.51	11.26	2.03
5	44,788.08	3,199.15	1,345.47	69.10	3

## 7. Conclusions

This paper introduced an end-to-end recommender system that determines which appliances should be turned on or off on specific time slots during the day and the associated readings of a household's power usage. In this work we present an end-to-end recommender system that generates recommendations on which appliances need to be turned on/off depending on the time of day and the respective power demand readings of a household. We first explore various energy disaggregation algorithms that can be used to perform non-intrusive load monitoring (NILM), in the absence of smart meters. Our benchmarking of two of the most popular and scalable algorithms with the UK-DALE dataset showed that the FHMM algorithm yields better results in disaggregating raw power meter data into appliance-level energy consumption. Then, using association rules and sequential pattern mining, we describe our clustering-based data engineering method for generating energy consumption profiles in households from fine-grained observations, which are then utilized to build appliance usage patterns. We also present our proof-of-concept prototype, used to demonstrate how such a system could be deployed to help users *via* an intuitive and interactive dashboard monitor their energy consumption, and also save energy. The prototype generates recommendations, while taking into consideration each household's energy consumption profile and customized preferences. Through experimental evaluation using the UK-DALE dataset collected from 4 very diverse households, in terms of suggestions and performance metrics, we found that *Apriori*, FP-Growth, and TRuleGrowth were relatively similar. TRuleGrowth has shown faster data processing and improved memory scalability. Depending on the number of appliances used in each household, our initial findings indicate that the system has a high recall rate and can help a household save between 2 and 17% of their energy. We intend to incorporate inter-appliance associations and correlations as part of our ongoing work to enhance the recommendations. We will also continue to assess how seasonality or time/day-specific profiles (such as weekdays vs. weekends) affect the quality of the recommendations and enhance energy savings. An interesting and relatively simple extension includes taking into consideration the current weather conditions, as an additional input signal in recommended actions (e.g., turn A/C off if the outside temperature drops below a threshold). Last but not least, based on the results of environmental psychology user studies, we plan to further improve the user interface by including information in the recommendations (such as money savings) that will also encourage users to make a change, or by letting them set saving goals and providing analytics about their actual gains as a reward for their action.

## Data availability statement

Publicly available datasets were analyzed in this study. This data can be found at: https://jack-kelly.com/data/.

## Author contributions

ME and IV contributed to the conception and design of the entire framework. IV identified the dataset and provided insights about the disaggregation process. ME supervised JD, AJ, AP, and AT in the implementation and prototype deployment process and helped them design the visualization or data analysis and experimental evaluation for this work. AT worked in pre- and post-processing the dataset. AP explored various algorithms for implementing energy disaggregation and compared their performances. AJ worked on resampling the dataset and visualization with t-SNE. In addition, he worked with JD to explore and apply various pattern mining algorithms to the dataset and helped with the design of the UI. JD also worked in implementing the UI of the system. All authors contributed to the article and approved the submitted version.

## Conflict of interest

The authors declare that the research was conducted in the absence of any commercial or financial relationships that could be construed as a potential conflict of interest.

## Publisher's note

All claims expressed in this article are solely those of the authors and do not necessarily represent those of their affiliated organizations, or those of the publisher, the editors and the reviewers. Any product that may be evaluated in this article, or claim that may be made by its manufacturer, is not guaranteed or endorsed by the publisher.
